# Molecular Traits Underlying the Growth Promotion and Metabolite Accumulation in *Rheum palmatum* Inoculated with Endophytic *Trichoderma citrinoviride* HT-1

**DOI:** 10.3390/ijms232113132

**Published:** 2022-10-28

**Authors:** Dawei Chen, Wenjing Shi, Yihan Wang, Jing Zhao, Hui Zhang, Lingyun Jia, Kun Sun

**Affiliations:** 1 College of Life Sciences, Northwest Normal University, Lanzhou 730070, China; 2 School of Basic Medical Sciences, Lanzhou University, Lanzhou 730000, China

**Keywords:** *Trichoderma citrinoviride*, *Rheum palmatum*, growth-promoting mechanisms, RNA-seq

## Abstract

*Trichoderma* spp. are an important plant-growth-promoting fungi. *Trichoderma citrinoviride* HT-1 was isolated from *Rheum palmatum* root, which has beneficial effects on growth and metabolite accumulation. However, the improvement mechanisms for growth and metabolite accumulation of *T. citrinoviride* HT-1 are unclear. In this study, RNA sequencing (RNA-seq) and high-performance liquid chromatography (HPLC) were used to measure the effect of different concentrations of conidial suspension of the HT-1 strain on the growth promotion and metabolite accumulation of *R. palmatum* seedlings. The results showed that the highest biomass and metabolites of *R. palmatum* seedlings were obtained through treatment with the HT-1 strain at a final spore concentration of 10^7^ spores/mL. RNA sequencing indicated that 1662 genes were upregulated and 2155 genes were downregulated after inoculation with 10^7^ spores/mL of the HT-1 strain. This strain induced significant upregulation of related genes in the phenylpropanoid biosynthesis pathway, plant hormone signal transduction pathway, biosynthesis of secondary metabolites pathway, and plant–pathogen interaction pathway in *R. palmatum*. The gene expression trends were revealed through quantitative real-time polymerase chain reaction (qRT-PCR) and were consistent with those determined by RNA-seq. Our results will help us to understand the growth-promoting mechanisms of the HT-1 strain on *R. palmatum* and provide a theoretical basis for the application of *T. citrinoviride* HT-1 as a biological fertilizer.

## 1. Introduction

With the increase in demand for natural products, food, medicine, energy, and other biotechnological aspects, excessive chemical fertilizers are used to increase the yield of plants [1,2]. However, the excessive use of chemical fertilizers has led to a series of environmental hazards, including soil pollution, decreased microbial diversity in soil, and harmful substance residues in food [3,4]. Therefore, it is necessary to find a safe and sustainable way to replace the application of chemical fertilizers to improve crop productivity and reduce environmental pollution. 

Endophytic fungi, which inhabit internal plant tissues, do not cause any symptoms of disease [5]. Many studies have reported that endophytic fungi can be used to improve the production of agricultural plants. Park et al. [6] reported that the inoculation of endophytic *T. citrinoviride* could significantly reduce *Panax ginseng* diseases caused by *Botrytis cinerea* and *Cylindrocarpon destructans*. Marcia et al. [7] found that photosystem II efficiency, leaf nitrogen, carbohydrate content, and the biomass of *Prosopis chilensis* were improved through the inoculation of endophytic *Penicillium*. Meanwhile, endophytes can affect the accumulation of metabolites in medicinal plants, which serve not only to store new bioactive metabolites, but also as a potential substitute for the metabolites of medicinal plants [8]. Stierle et al. [9] first reported that endophytic *Taxomyces andreanae* was isolated from *Taxus brevifolia*, which could produce taxol and related compounds. Therefore, endophytes play an important role in promoting the growth, and regulating the accumulation, of metabolites in medicinal plants, and are a potential source for the substitution of medicinal plants metabolites.

*Rheum palmatum* is a famous traditional Chinese medicine (TCM) [10], which has been used for medical purposes and cultivation by the Chinese for thousands of years [11]. The *Divine Farmer’s Herb-Root Classic* reported that it can be used to treat many diseases, such as constipation, jaundice, ulcers, and gastrointestinal hemorrhage [12]. The root contains rich anthraquinones, including aloe-emodin, rhein, emodin, chrysophanol, and physcion, which has been widely studied due to its excellent biological activity. Anthraquinones have many properties, such as anti-inflammatory, antitumor, antiviral, antiobesity, and anticoagulant effects [13]. However, the wild resources of *Rheum* species have been shrinking faster than ever in recent years to meet the commercial demand for this species, which has led to the destruction of most natural populations [13]. Efforts have been made to develop a sustainable and environmentally friendly way of improving yield and quality.

*Trichoderma citrinoviride* HT-1 was isolated from *R. palmatum* root, which can promote the growth of pakchoi (*Brassica chinensis* L.) [14]. However, the growth-promoting mechanisms of *T. citrinoviride* HT-1 are still unknown. Previous researchers studied a single or specific category of metabolites associated with the growth promotion of plants [15]. During the process of endophytic fungi promoting, the growth and metabolite accumulation of plants lacked sufficient systematic research about their molecular mechanisms. In the present study, RNA-seq and HPLC were used to explore the growth-promoting mechanisms of endophytic *T. citrinoviride* HT-1 on *R. palmatum*, and these results lay a foundation for the development of biological agents.

## 2. Results

### 2.1. Effect of T. citrinoviride HT-1 on Growth of Seedlings

**Figure 1:**
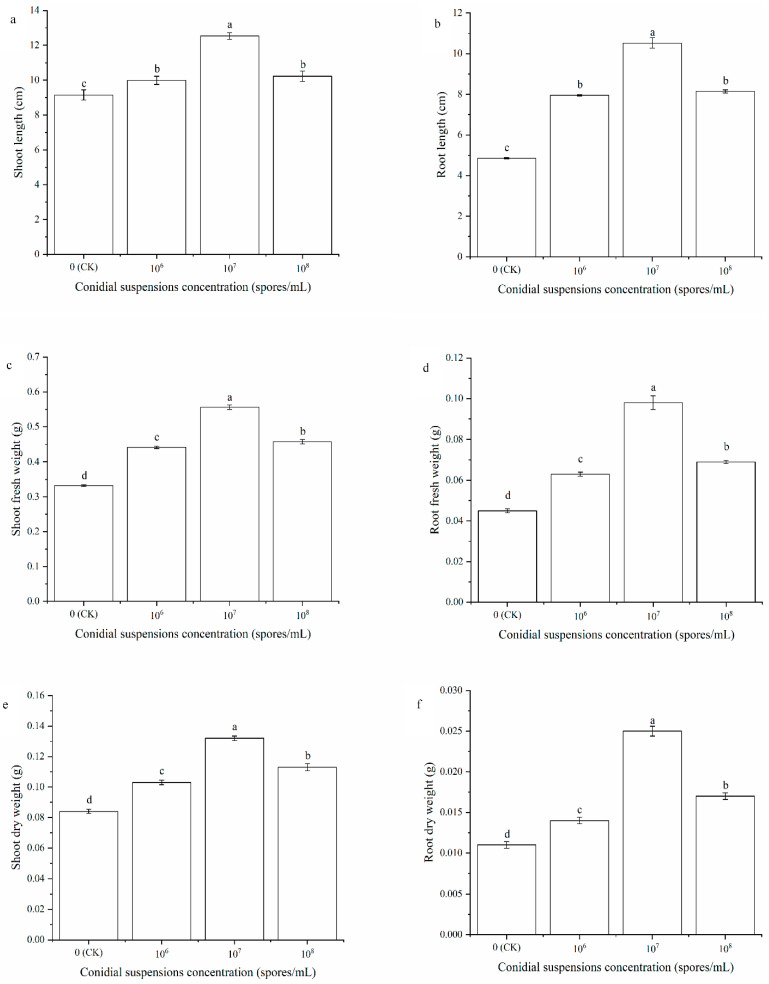
Effects of different concentration conidial suspensions of HT-1 strain on shoot length (**a**), root length (**b**), shoot fresh weight (**c**), root fresh weight (**d**), shoot dry weight (**e**), and root dry weight (**f**) of *R. palmatum* under greenhouse. Values are mean ± SD (*n* = 15 plants). Different letters above the bars indicate the differences are significant at *p* < 0.05.

**Figure 2:**
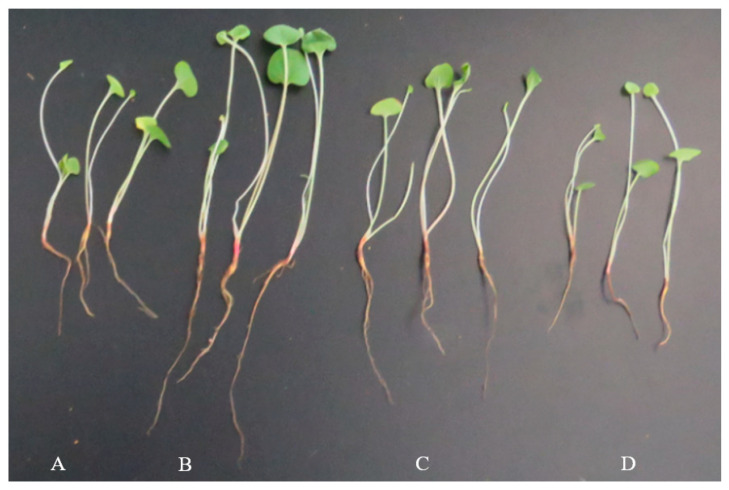
Effects of different concentration conidial suspensions of HT-1 strain on the growth of seedlings. Note: (**A**), seedlings treated with HT-1 strain at spore concentration of 10^6^ spores/mL; (**B**), seedlings treated with the HT-1 strain at spore concentration of 10^7^ spores/mL; (**C**), seedlings treated with HT-1 strain at spore concentration of 10^8^ spores/mL; (**D**), CK (seedlings treated with distilled water).

In the greenhouse experiment, treatment with different conidial suspension concentrations of HT-1 strain showed an obvious improvement in the growth of *R. palmatum* (Figure 1 and Figure 2). Treatment with the HT-1 strain at a spore concentration of 10^7^ spores/mL showed the highest values for shoot length (12.54 cm, Figure 1a) and root length (10.52 cm, Figure 1b), which increased by 3.39 cm and 5.67 cm, and 37.05% and 116.91%, respectively, compared with the control. A significant difference was observed at *p* < 0.05.

Positive effects on the biomass of *R. palmatum* were observed with the application of different conidial suspension concentrations of the HT-1 strain (Figure 1). Treatment with the HT-1 strain at a spore concentration of 10^7^ spores/mL showed the highest shoot fresh weight (0.556 g/plant, Figure 1c), root fresh weight (0.098 g/plant, Figure 1d), shoot dry weight (0.132 g/plant, Figure 1e), and root dry weight (0.025 g/plant, Figure 1f), which increased by 67.47%, 117.78%, 57.14%, and 127.27%, respectively, compared with the control. A significant difference was observed at *p* < 0.05.

### 2.2. Effect of T. citrinoviride HT-1 on Root Activity of Seedlings

**Figure 3:**
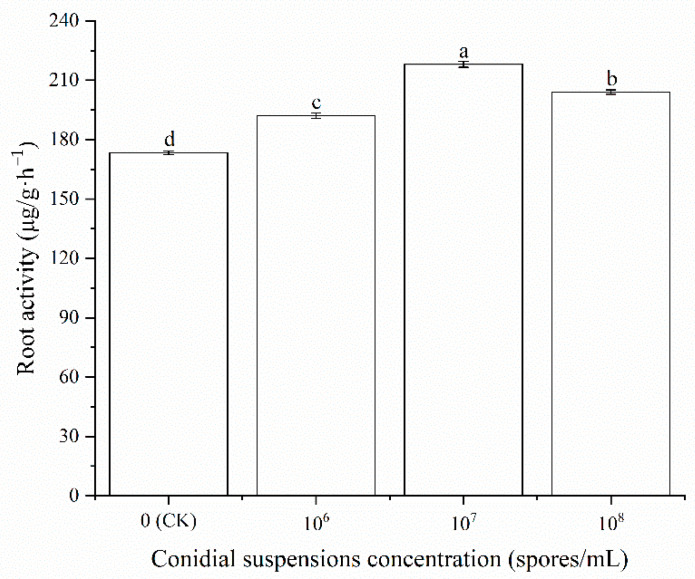
Effects of different concentration conidial suspensions of HT-1 strain on the root activity of *R. palmatum.* Values are mean ± SD (*n* = 15 plants). Different letters above the bars indicate the differences are significant at *p* < 0.05.

As shown in Figure 3, different concentrations of HT-1 strain conidial suspensions significantly increased the root activity of *R. palmatum.* The highest root activity (218.09 μg/g·h^−1^) in *R. palmatum* was obtained under the treatment of the HT-1 strain at a spore concentration of 10^7^ spores/mL, which was increased by 25.77% compared with the control. There were significant differences at the 0.05 level (*p* < 0.05).

### 2.3. Effect of T. citrinoviride HT-1 on Metabolites of Seedlings

**Figure 4:**
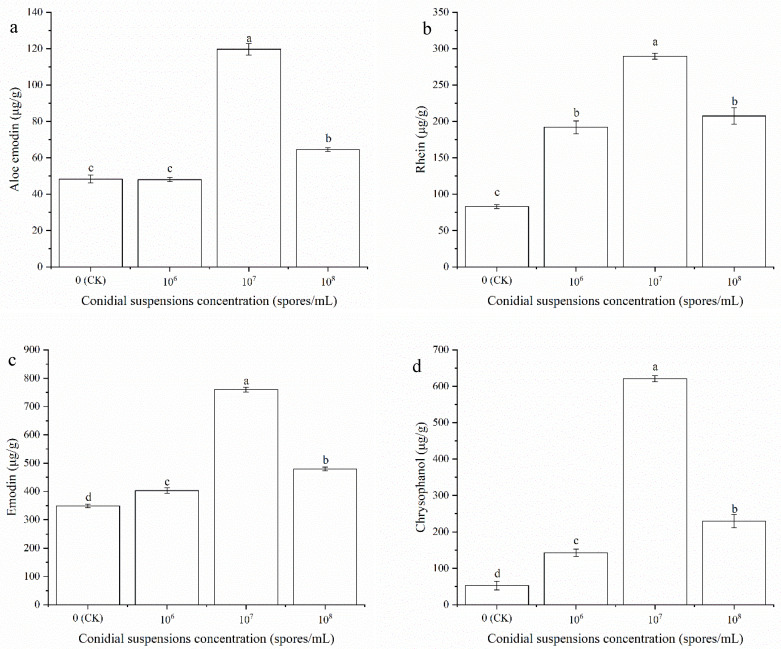
Effects of different concentration conidial suspensions HT-1 strain on metabolites accumulation in *R. palmatum*. (**a**) Aloe emodin; (**b**) Rhein; (**c**) Emodin; (**d**) Chrysophanol. Values are mean ± SD (*n* = 5 replicates). Different letters above the bars indicate the differences are significant at *p* < 0.05.

As shown in Figure 4, different concentrations of HT-1 conidial suspensions increased the metabolites of *R. palmatum.* The highest aloe-emodin (119.70 μg/g), rhein (289.75 μg/g), emodin (759.86 μg/g), and chrysophanol (621.03 μg/g) contents were tested under the treatment of the HT-1 strain at a spore concentration of 10^7^ spores/mL, which increased by 71.30, 206.66, 411.13, and 568.31 μg/g compared with the control, respectively. There was a significant difference at the *p* < 0.05 level.

### 2.4. Unigene Annotation and Identification of DEGs

A total of 232,520,004 and 286,519,896 raw reads, respectively, were obtained from the control (CK) and treatment (HT-1 strain) group. However, a total of 226,702,706 (CK) and 278,833,662 (HT-1 strain) were retained after assembly (Table 1). Sequencing data were submitted to the NCBI Sequence Reads Archive database with the SRA accession number PRJNA751164. A total of 213,797 unique sequences were annotated based on BLASTX alignment searches of five public databases, including GO, KEGG, NR, Swiss-Prot, and Pfam. 

**Figure 5:**
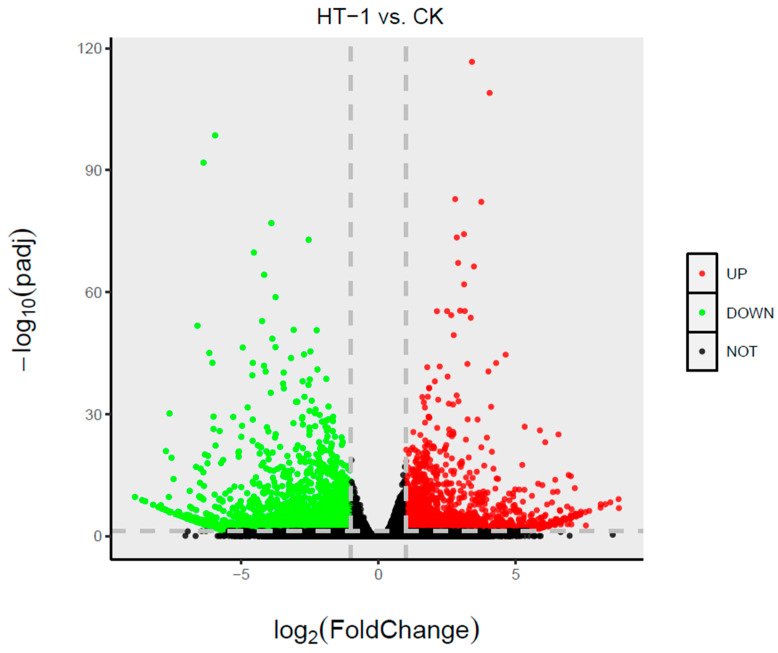
Volcano plots of differentially expressed genes. HT-1, *R. palmatum* roots from group the treated by 10^7^ spores/mL HT-1 strain; CK, *R. palmatum* roots from the control group.

Compared with the *R. palmatum* grown in control soil, *R. palmatum* was grown in soil treated with 10^7^ spores/mL HT-1 strain for 90 d. A total of 1662 genes were upregulated (logFC > 1), and 2155 genes (|logFC| > 1) were downregulated (Figure 5).

### 2.5. GO Function and KEGG Pathway Enrichment Analysis of the DEGs

**Figure 6:**
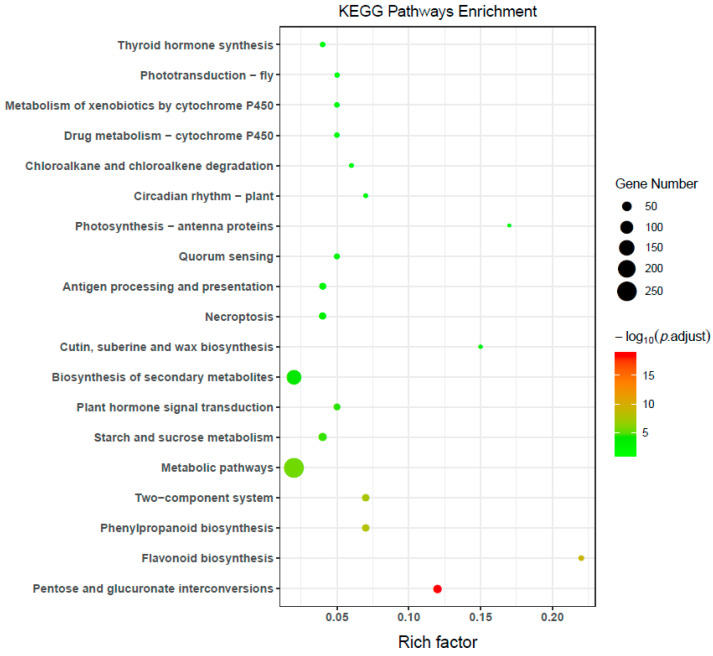
Kyoto Encyclopedia of Genes and Genomes (KEGG) enrichment analysis of differentially expressed genes (DEGs).

The KEGG pathway database includes information about molecular interactions in known metabolic and regulatory pathways. The KEGG database was used to perform enrichment analysis of the DEGs in the roots of *R. palmatum*; Figure 6 is a visual presentation of the KEGG enrichment analysis results. The degree of KEGG enrichment is measured by the Rich factor, *p*.adjust, and number of genes enriched in this pathway. The Rich factor refers to the ratio of the number of DEGs in the pathway to the total number of all annotated genes in the pathway. The larger the Rich factor, the greater the degree of enrichment. The *p*.adjust is the *p*-value after correction for multiple hypothesis testing. The more significant the enrichment, the smaller the value of −log10 (*p*.adjust). KEGG pathway enrichment analysis showed that DEGs were significantly matched in the KEGG database and were enriched in 241 pathways; the distribution of the DEGs in five major categories was performed using the KEGG database. Through analysis, we found that 26, 22, 134, and 17 genes, respectively, were enriched in the phenylpropanoid biosynthesis pathway, plant hormone signal transduction pathway, biosynthesis of secondary metabolites pathway, and plant–pathogen interaction pathways (Figure 6). Those pathways were related to growth, metabolite accumulation, and response to stress. 

**Figure 7:**
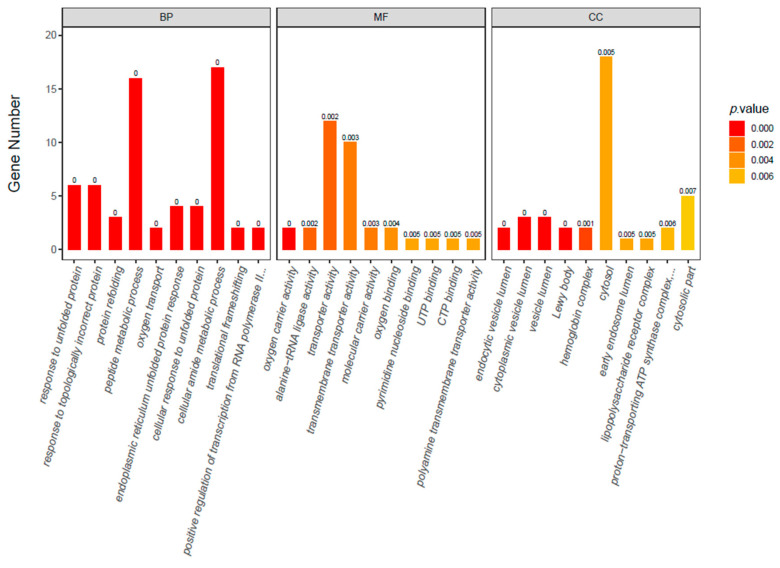
Chart summarizing the results of Gene Ontology (GO) enrichment analysis.

GO is an international standardized gene function classification system that describes the properties of genes and their products in any organism. The top 30 significant GO enrichment terms are displayed to evaluate the DEGs in terms of their predicted functions (Figure 7). The DEGs were assigned to three main GO categories: biological processes, cellular components, and molecular functions. In the biological process category, functions such as peptide metabolic process and cellular amide metabolic process were detected for the significant terms, along with multiple genes of both. In the cellular component category, functions such as transporter activity and transmembrane transporter activity were the significant terms, and the gene numbers of both were multiple. For the molecular functions category, cytosol was the most significant term, with multiple numbers of genes.

### 2.6. Verification of Partial DEGs Using RT-qPCR

**Figure 8:**
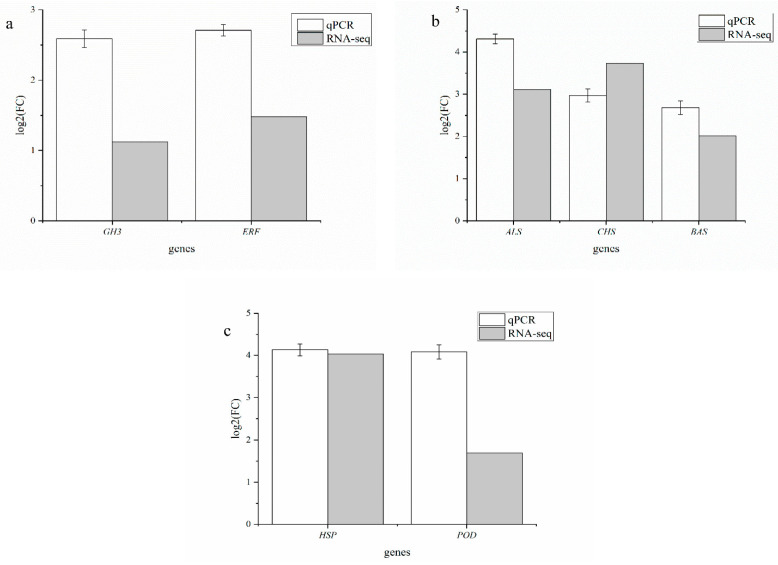
qRT-PCR assay of genes in *Rheum palmatum.* qRT-PCR verified the accuracy of the transcriptome data. (**a**) qRT-PCR results of genes related to plant hormone transduction in *R. palmatum*. (**b**) qRT-PCR results of genes related to biosynthesis of secondary metabolites in *R. palmatum.* (**c**) qRT-PCR results of genes related to phenylpropanoid biosynthesis and plant–pathogen interaction in *R. palmatum*. Error bars indicate the SD from the mean.

qRT-PCR is one of the important methods used to verify the gene expression level in RNA-seq. To verify the accuracy of the RNA-seq data, we selected some DEGs in *R. palmatum* for qRT-PCR (Table 2). The RNA-seq of *R. palmatum* was treated with 10^7^ spores/mL; The HT-1 strain revealed that genes related to plant hormone transduction (Gretchen Hagen 3 (*GH3*) and ethylene response factor (*ERF*)) were upregulated by 1.13 times and 1.48 times, respectively, compared with the control, distilled water (CK). qRT-PCR showed that the expression of *GH3* (TR2359_c0_g1) and *ERF* (TR6369_c0_g1) was upregulated by 2.59 and 2.71 times, respectively (Figure 8a). The gene expression trends that were demonstrated by qRT-PCR were consistent with those determined by RNA-seq. In RNA-seq, three genes (aloesone synthase (*ALS*), chalcone synthase (*CHS*), and benzalacetone synthase (*BAS*)) related to the biosynthesis of secondary metabolites were upregulated by 3.11, 3.73, and 2.01 times, respectively, compared with the CK. qRT-PCR indicated that the expressions of *ALS* (TR407_c0_g1), *CHS* (TR134901_c0_g1), and *BAS* (TR407_c2_g2) were upregulated by 4.31, 2.97, and 2.68 times, respectively (Figure 8b). The results of RNA-seq indicated that two genes related to plant–pathogen interaction and phenylpropanoid biosynthesis (heat shock protein (*HSP*) and peroxidase *(POD*)) were upregulated by 4.03 times and 1.69 times, respectively, compared with the CK. qRT-PCR indicated that the expression of *HSP* (TR61073_c0_g1) and *POD* (TR21_c0_g2) were upregulated by 4.13 and 4.08 times, respectively (Figure 8c).

## 3. Discussion

In our study, *T. citrinoviride* HT-1 significantly promoted the growth and metabolite accumulation of *R. palmatum* seedlings. Many studies have reported the growth-promoting effects of *Trichoderma* spp. [16]. However, limited studies have investigated the global variation in plants during their growth. To the best of our knowledge, this is the first study to reveal the effect of *T. citrinoviride* HT-1 on *R. palmatum* roots during the growth-promotion process, including the many pathways related to secondary metabolism.

We analyzed the entire transcriptome and differential gene expression in *R. palmatum* seedlings after treatment with the HT-1 strain. We observed significant changes in a total of 1662 genes that were upregulated and in 2155 downregulated genes. The GO and KEGG analysis results of DEGs showed significant differences that indicated that the HT-1 strain can significantly promote the growth of *R. palmatum* seedlings. Our results also indicated that the HT-1 strain regulates different pathways to promote the growth of *R. palmatum*.

The KEGG pathway database contains information about molecular interactions in known metabolic and regulatory pathways [17]. KEGG enrichment analysis revealed that a large number of genes are involved in pathways related to phenylpropanoid biosynthesis, secondary metabolism, plant pathogens, and hormone signaling transduction, amongst others. These pathways are principally involved in cell wall biosynthesis, nutrient accumulation, secondary metabolism, and hormone signaling [18]. This analysis also showed that the metabolic pathways and biosynthesis of amino acids are significantly regulated in response to the HT-1 strain. A previous study reported that amino acids not only participate in protein synthesis, but also play an important role in the growth, development, reproduction, defense, and environmental responses of plants [19]. These pathways have received great attention for being the most important characteristic of medicinal plants for biomass production and the accumulation of secondary metabolites.

The transcriptome of *R. palmatum* roots during their interaction with the HT-1 strain revealed that the HT-1 strain can stimulate auxin and ethylene signal transduction in *R. palmatum*. *GH3* is involved in the different growth and developmental processes of plants and can catalyze adenylation of indole-3-acetic acid, jasmonic acid, and salicylic acid [20]. The *ERF* is one of the main transcription factor protein groups in the plant kingdom and is potentially involved in the growth and stress responses of various plants [21]. DEG analysis showed that *GH3* and *ERF* were upregulated by 1.13–1.98 times and 1.12–1.93 times, respectively, compared with those in *R. palmatum* roots not cultured with the HT-1 strain. These results are similar to those reported by Liu et al. [22], who concluded that genes (*GH3*) related to plant hormone transduction were upregulated by 1.51–4.25 times, compared with those in tobacco roots not cultured with *Paenibacillus polymyxa* YC0136. 

Anthraquinone is a well-known secondary metabolite in *R. palmatum* [11]. Owing to the importance of anthraquinones in the field of medicine and biology, efficiently and quickly obtaining beneficial quinones and improving the synthesis efficiency of plant anthraquinones have received more attention. Therefore, it is crucial to study the metabolism mechanisms of anthraquinones in plants for their efficient production. Many studies have reported that the synthesis of anthraquinones is very complex, and the synthesis of precursors involves multiple metabolic pathways [23]. Limited information is available on the biosynthesis of anthraquinones in plants, and, to the best of our knowledge, there are only two reports on anthraquinone-synthesis-related enzymes in *Polygonum cuspidatum* [24,25]. Anthraquinones may also be biosynthesized through the polyketide pathway in plants [26]. Chalcone synthase (*CHS*), also called polyketide synthase III (PKS III), is a key enzyme involved in the polyketide pathway, and the involvement of polyketide synthase III and UDP-glycosyltransferase in anthraquinone formation was proven [26]. *ALS* and *BAS* are members of the PKS III family and have been reported to be involved in anthraquinone formation [27,28]. In this study, three genes (*ALS*, *CHS*, and *BAS*) related to the biosynthesis of secondary metabolites were upregulated compared with those in *R. palmatum* roots not treated with the HT-1 strain, indicating that the HT-1 strain induced upregulation of anthraquinone-synthesis-related genes. 

Furthermore, we found that phenylpropanoid biosynthesis plant–pathogen-interaction-related genes of *R. palmatum* were significantly upregulated after HT-1 strain treatment through transcriptome analysis. Peroxidase (*POD*) is an antioxidant enzyme that is an integral component of the oxidative defense system [29]. Heat shock protein (*HSP*) is crucial for the development of, and responses to, diverse stresses in plants [30]. These results indicate that the HT-1 strain can improve the ability of *R. palmatum* to respond to stress. *R. palmatum* may activate the defense mechanism after treatment with the HT-1 strain, and then form symbionts with the HT-1 strain, which can increase the synthesis of antioxidants, restore the balance between substance synthesis and energy metabolism, and improve the survival ability of plants.

Due to the complexity of plant–microorganism interactions, research on them is scarce, especially for medicinal plants. The identification of plant-growth-promoting microorganism strains that are associated with this crop and promote its growth is of great importance. Large numbers of microorganisms exist in nature, and the identification of microorganisms that may increase plant growth and yield is fundamental to increasing the sustainability of agricultural systems. While fertilizers and pesticides increase crop yields, they are also associated with negative effects. With extensive use of biopromoters or biofertilizers, environmental pollution can be reduced with the added benefit of ensured food safety.

## 4. Materials and Methods

### 4.1. Plant Samples and Fungi Culture

The seeds of *R. palmatum* were collected from Li County (2282 m, 104°52′38.03″ E, 34°05′46.48″ N) in Gansu province, China. *T. citrinoviride* HT-1 (Accession number: MT781604.1) was isolated from *R. palmatum* roots from HuaTing city (1746 m, 106°30′26.28″ E, 35°18′22.18″ N) in Gansu province, China, which was saved in the Microbial Collection of College of Life Science, Northwest Normal University. The HT strains were kept on potato dextrose agar (PDA) medium under 4 °C. The strain was grown on PDA plates at 28 °C for 7 d and a 16 h light/8 h dark cycle. Conidial suspensions were obtained from a PDA plate with 2 mL sterile water, and diluted to 10^6^, 10^7^, and 10^8^ spores/mL for the next experiments.

### 4.2. Test for Capacity to Promote Plant Growth (Pot Experiment)

*R. palmatum* seeds were selected according to their size and color, 2% (*v*/*v*) NaClO solution was utilized to surface-sterilize for 20 min at room temperature, and they were then rinsed completely with deionized water. Finally, the disinfected seeds were incubated in sterilized Petri dishes (diameter = 90 mm), with two layers of filter paper at 25 °C for 4 d. The seedlings were sown in plastic pots (25 × 25 cm, 10 seedings per pot) and filled with a sterilized soil/vermiculite (3:1, *v*/*v*) mixture after seed germination. Half-strength Hoagland’s nutrient solution (including 2 mM KNO_3_, 0.5 mM NH_4_H_2_PO_4_, 0.1 mM Ca(NO_3_)_2_·4H_2_O, 0.25 mM MgSO_4_·7H_2_O, 0.5 mM Fe citrate, 92 μM H_3_BO_3_, 18 μM MnCl_2_·4H_2_O, 1.6 μM ZnSO_4_·7H_2_O, 0.6 μM CuSO_4_·5H_2_O, and 0.7 μM (NH_4_)_6_Mo_7_O_24_·4H_2_O) was irrigated every 3 d and maintained the soil water content at 60–70%. The pots were cultured under temperature of 25 ± 1 °C, relative humidity of 70–75%, 14 h light/10 h dark cycle, a light intensity of 120 μmol/m^2^/s, and watered every 2 d. After growth for 2 weeks, uniform seedlings were selected, and the seedlings rhizosphere was irrigated with 50 mL of different treatments (10^6^, 10^7^, and 10^8^ spores/mL) and CK (control, distilled water); fifty biological replicates were selected for each treatment.

### 4.3. Plant Growth Measurement

The plant biomass was determined after growing for 90 d. The attached soil from the plant root surface was removed by tap water, and then rinsed thrice with deionized water. The plants were divided into roots and shoots to measure biomass parameter; fifteen biological replicates were selected for measurement.

The root activity of *R. palmatum* was tested according to the triphenyltetrazolium chloride (TTC) method [31]. The root activity was measured through TTC, was reduced to insoluble red triphenylformazan. A total of 0.1 g of fresh roots was cut into pieces, and then soaked in 0.6% (*w*/*v*) TTC solution (TTC was dissolved in phosphate buffer at pH 7.0) in the dark at 30 °C for 24 h. Then, the roots were rinsed twice, filter paper was used to remove the water from the roots’ surfaces, and the roots were immersed in 95% (*v*/*v*) ethanol for 30 min at room temperature. The absorbance values of the extraction solutions were tested by a spectrophotometer (Hitachi U3010, Tokyo, Japan) at 485 nm.

### 4.4. Metabolites of R. palmatum Quantitative Analysis

Standard aloe-emodi, rhein, emodin, chrysophanol, and physcion were obtained from Shanghai R&D Center for Standardization of Traditional Chinese Medicines. High-performance liquid chromatography (HPLC)-ultrapure water, analytical-grade methanol, NaHCO3, and phosphoric acid were purchased from Sangon Biotech, Ltd. (Shanghai, China). 

The metabolites of *R. palmatum* were measured according to the methods of Chen et al. [32]; the dried root of different treatments (five replicates) was pulverized and sieved by using a mesh (300 μm). An amount of 0.15 g of powder was precisely weighed, 10 mL 0.1% (*w*/*v*) NaHCO_3_ solution was added, and ultrasonic treatment was performed (30–40 °C, 700 W) for 20 min. Then, 40 mL of methanol was added and treated using ultrasound for 50 min. The sample solution was filtered by a Millipore filter unit (0.22 μm), and then 10 μL of sample solution was injected into the HPLC for measurement. 

HPLC system and parameters were set according to the methods of Chen et al. [33]; the metabolites content of samples was determined by HPLC (Waters E2695, Milford, MA, USA) using C18 (4.6 × 250 mm, 5.0 μm) at 30 °C. The mobile phase of HPLC was methanol −0.1% phosphoric acid (80:20); the flow rate was 1 mL/min. The detection wavelength was 254 nm.

### 4.5. Total RNA Extraction and Transcriptomic Analysis

The treatment of best growth-promoting and CK (control, distilled water) were selected for transcriptomic analysis. Total RNA from *R. palmatum* roots was extracted and purified using TRIzol reagent (Invitrogen, Thermo Fisher Scientific Inc., Waltham, MA, USA). The quality of RNA was determined by using an Agilent 2100 Bioanalyzer (Agilent Technologies, Palo Alto, CA, USA) and a NanoDrop (Thermo Fisher Scientific Inc., Waltham, MA, USA). The RNA integrity numbers (RINs) of RNAs > 7 were selected to build the library. RNA sequencing library preparations were built according to the manufacturer’s instructions (NEBNext^®^Ultra^TM^ RNA Library Prep Kit for Illumina^®^, NEW ENGLAND Biolabs^®^ Inc., Ipswich, MA, USA). Libraries with different indices were multiplexed and loaded on an Illumina HiSeq instrument according to the manufacturer’s instructions (Illumina, San Diego, CA, USA). RNA sequencing was performed using a 2 × 150 bp paired-end configuration. Image analysis and base calling were performed using HiSeq Control Software (HCS) + OLB + GAPipeline-1.6 (Illumina) on the HiSeq instrument.

Raw data were reserved in fastq file format. Low-quality data and adaptor sequences were filtered out to ensure the accuracy of the data. De novo assembly was performed to generate transcripts according to the Trinity method [34]. HTSeq (v0.6.1) was used to estimate the gene and isoform expression levels from paired-end clean data [35]. Differentially expressed genes (DEGs) were analyzed using DESeq2 (V1.6.3) in the Bioconductor software package [36]. Kyoto Encyclopedia of Genes and Genomes (KEGG) pathway enrichment analysis of DEGs was performed using KOBAS [37]. Gene Ontology (GO), an international standardized gene function classification, was performed using the BLAST2 GO tool [38].

### 4.6. qRT-PCR Assay for DEGs in RNA-Seq

An amount of 1 μg of purified total RNA was used as a template for first-strand cDNA synthesis using an Evo M-MLV RT Kit with gDNA Clean for qPCR (Accurate Biotechnology (Hunan) Co., Ltd., Changsha, Hunan province, China). Several genes identified through RNA-seq were selected for amplification using SYBR Green qPCR. Primers were designed using Beacon Designer 7 (listed in Table 3). Actin was used as a reliable internal reference gene for judging the efficiency of the RT-PCR system and the quality of RNA extracts. Three biological replicates were selected for measurement, and the genes’ expression level were calculated according to the method of 2^−ΔΔCT^ [39].

### 4.7. Statistical Analysis

All data were analyzed by using SPSS16.0 software for variance (one-way ANOVA) and Duncan’s multiple range test (*p* < 0.05). Columns were constructed by using Origin 9.1 (Origin Software, Northampton, MA, USA).

## 5. Conclusions

In the present study, we described the *R. palmatum* growth-promoting and metabolite accumulation effect of *T. citrinoviride* HT-1, an endophytic fungus that was previously isolated from the roots of *R. palmatum*. The RNA-seq results indicated that the growth-promoting and metabolite accumulation effect of the HT-1 strain was associated with the upregulated expression of genes implicated in phenylpropanoid biosynthesis, plant hormone signal transduction, biosynthesis of secondary metabolites, and plant–pathogen interaction pathways. The research provided an in-depth understanding of the mechanism of action of the endophytic *T. citrinoviride* HT-1 in relation to *R. palmatum* growth promotion and metabolite accumulation.

## Figures and Tables

**Table 1:** Summary of trimming and read mapping results of the sequences obtained from *Rheum palmatum* roots treated without or with HT-1 strain inoculation.

Sample	Raw_Reads	Clean_Reads
CK-1	67,794,322.00	66,127,198.00
CK-2	99,675,100.00	97,372,554.00
CK-3	65,050,582.00	63,202,954.00
HT-1-1	97,036,064.00	94,274,502.00
HT-1-2	84,968,252.00	82,706,638.00
HT-1-3	104,515,580.00	101,852,522.00

**Table 2:** The annotation of selected functional genes.

Gene Name	Gene ID	NR Annotation
*GH3*	TR2359_c0_g1	Indole-3-acetic acid-amido synthetase GH3
*ERF*	TR6369_c0_g1	Ethylene-responsive transcription factor 1B
*BAS*	TR407_c2_g2	Polyketide synthase BAS, benzalacetone synthase
*CHS*	TR134901_c0_g1	Chalcone synthase
*ALS*	TR407_c0_g1	Aloesone synthase
*HSP*	TR61073_c0_g1	Heat shock protein
*POD*	TR21_c0_g2	Peroxidase

**Table 3:** qRT-PCR primers used in this study.

Gene	Primer Name	Primer Sequence
*actin*	*actin*-F	5′-GACAGTCGTTCTCAGGCAAGGC-3′
	*actin*-R	5′-GACGAGAAGAAACGGGAGGTTGTG-3′
*HSP*	*HSP*-F	5′-CGATTGGGAAGATCATTTGGCTGT-3′
	*HSP*-R	5′-TCAAATGGTGCACGTTTTGGTACA-3′
*POD*	*POD*-F	5′-TCCTCGGCGGCATAACCTACTC-3′
	*POD*-R	5′-TGGAGAAGCTCGATTGGAGTTGTTG-3′
*ALS*	*ALS*-F	5′-TAGCCCTTGTGCCTTCTCCGATAG-3′
	*ALS*-R	5′-GAGCGAGAGCATGGCAGATTGG-3′
*CHS*	*CHS*-F	5′-AGGAAGTCGGTGGAGGAAGGTATG-3′
	*CHS*-R	5′-CTTCGCAGCACAACGGTTTCAAC-3′
*BAS*	*BAS*-F	5′-CAACCACTGGAGAAGGGCTTGAG-3′
	*BAS*-R	5′-GGTACACTGCGTAGCACCACAG-3′
*GH3*	*GH3*-F	5′-CCAGACAACAACTCCTCAACACCTC-3′
	*GH3*-R	5′-GCTACGCTGACACCAAGACCATC-3′
*ERF*	*ERF*-F	5′-CAACCACCGCATCCTACAACTCC-3′
	*ERF*-R	5′-CTTGACACGCACCTCCGATTCC-3′

## Data Availability

The transcriptome data used in this manuscript were submitted to the NCBI and the Accession number was PRJNA751164 (https://www.ncbi.nlm.nih.gov/sra (accessed on 31 July 2021).
